# Draft genome sequencing of an *Enterococcus faecium* strain isolated from healthy cat (Turkish Van) in Bangladesh

**DOI:** 10.1128/mra.01263-25

**Published:** 2025-12-23

**Authors:** Muhammad Abdul Mannan, Sarah Binte Hossain, Md. Morshedur Rahman, Kh. Yeashir Arafat, Kazi Mohammad Rokan Uddin, Md. Abul Hashem, Md. Rezaul Karim, M. Nazmul Hoque

**Affiliations:** 1 Department of Microbiology and Parasitology, Faculty of Animal Science and Veterinary Medicine, Sher-e-Bangla Agricultural University 54494, Dhaka, Bangladesh; 2 Molecular Biology and Bioinformatics Laboratory, Department of Gynecology, Obstetrics and Reproductive Health, Gazipur Agricultural University 198780, Gazipur, Bangladesh; 3 Molecular Biology and NGS Laboratory, Chattogram Veterinary and Animal Sciences University 130057, Chattogram, Bangladesh; 4 Animal Health Research Division, Bangladesh Livestock Research Institute (BLRI) 560480, Dhaka, Bangladesh; 5 Department of Cell and Developmental Biology, Northwestern University, Feinberg School of Medicine 12244, Chicago, Illinois, USA; University of Maryland Baltimore, Baltimore, Maryland, USA

**Keywords:** draft genome, *E. faecium*, healthy cat, public health

## Abstract

*Enterococcus faecium* is a lactic acid bacterium of both pathogenic and probiotic relevance with important public health implications. We report the draft genome of *E. faecium* strain SBH-SAU.Ef.2025 isolated from the nasal swab of a healthy cat. The genome is 2,772,570 bp in length, comprising 159 contigs with 38.0% GC content.

## ANNOUNCEMENT

*Enterococcus faecium* is a gram-positive, facultatively anaerobic, gamma-hemolytic, or non-hemolytic lactic acid bacterium that is widely distributed in nature. It colonizes the gastrointestinal tract and other organs of humans and animals and can also be isolated from plants, soil, freshwater, marine environments, and food (1–3). This species exhibits diverse characteristics, functioning as an opportunistic pathogen or a probiotic, and carries virulence and antimicrobial gene clusters, which underscores its significance for public health, food safety, and zoonotic transmission (2, 4). In this study, the *E. faecium* strain SBH-SAU.Ef.2025 was isolated from the nasal swab of a healthy cat (Turkish Van) in Dhaka, Bangladesh (23.79°N, 90.30°E) to characterize their genetic features and assess their potential impact on animal and human health.

A nasal sample from a Turkish Van cat was collected using a sterile cotton swab and immediately transferred to the laboratory in a sterile tube containing PBS at 4°C for further processing and investigation. Within 4 h, the swab was enriched overnight in nutrient broth (Oxoid, UK) at 37°C under aerobic conditions, followed by a 10⁻³ serial dilution, and a loopful of the diluted broth was streaked onto *Streptococcus* selective agar (HiMedia, India) for incubation at 37°C for 24 h. Individual colonies were then subcultured onto fresh agar plates at 37°C for 24 h to obtain pure isolates. The phenotypic characterization of *E. faecium* isolates was conducted based on colony morphology (characterized by translucent, glass-like colorless colonies) and gram staining (indicating gram-positive and cocci). Genomic DNA was extracted from nutrient broth cultures prepared by inoculating purified colonies and incubating at 37°C using the QIAamp DNA Mini Kit (QIAGEN, Germany) (5, 6). DNA sequencing library was prepared from 1 ng of DNA using the Nextera DNA Flex Kit (Illumina, USA) (7) and sequenced on the Illumina MiSeq platform with paired-end reads of 2 × 250 bp, generating a total of 1,795,600 reads (SRR35925284) and an average sequencing depth of ~300× (8, 9). The raw sequence data were quality-checked using FastQC v0.11.3 (10). Following this, Illumina adapters, known artifacts, and phiX reads were removed using Trimmomatic v0.39.2 (11). The cleaned paired-end reads were assembled with SPAdes v4.0.0 (12), resulting in a 2,772,570 bp genome (2.8 Mb) comprising 159 contigs with a GC content of 38%. Genome annotation using the NCBI Prokaryotic Genome Annotation Pipeline (PGAP v6.10) (13) predicted 2,804 genes, including 2,749 coding sequences and 55 RNAs (six rRNAs, 45 tRNAs, and four ncRNAs) (Table 1). The assembled genome contigs were screened for virulence factors using the core data set of the Virulence Factor Database (VFDB v6.0) (14) and for antimicrobial resistance genes (ARGs) using the Comprehensive Antibiotic Resistance Database (CARD v3.2.4) (15), applying thresholds of 80% coverage and 80% nucleotide identity. Two *scm* genes encoding collagen-binding adhesins were identified as virulence factors in strain SBH-SAU.Ef.2025. Additionally, five ARGs were detected, including two copies of the *vanY* gene within the *vanB* cluster, along with *efmA*, *efrA*, and *AAC(6′)-Ii*. Default parameters were used for all software, unless otherwise specified.

**TABLE 1:** Genomic features of *E. faecium* strain *SBH-SAU.Ef.2025* isolated from the nasal swab of a healthy cat

Genomic features	*E. faecium* SBH-SAU.Ef.2025
GenBank accession	JBRIQE000000000
BioSample accession	SAMN51821915
Assembled genome size (bp)	27,72,570
GC %	38
Coverage (×)	300
N_50_ value (bp)	80,256
Number of contigs	159
Genes (total)	2,804
CDSs (total)	2,749
Genes (coding)	2,663
CDSs (with protein)	2,663
Genes (RNA)	55
rRNAs	2, 1, 3 (5S, 16S, 23S)
Complete rRNAs	1, 1, 1 (5S, 16S, 23S)
Partial rRNAs	1, 2 (5s, 23s)
tRNAs	45
ncRNAs	4
Pseudo genes (total)	86
CDSs (without protein)	86
Pseudo genes (ambiguous residues)	0 of 86
Pseudo genes (frameshifted)	33 of 86
Pseudo genes (incomplete)	47 of 86
Pseudo genes (internal stop)	21 of 86
Pseudo genes (multiple problems)	12 of 86
Number of ARGs	05
Number of VFGs	02

## Data Availability

The Whole Genome Shotgun project of the *E. faecium* strain SBH-SAU.Ef.2025 has been deposited in GenBank and the NCBI Sequence Read Archive (SRA) under accession SRR35925284 and BioProject accession number PRJNA1333976. The version described in this paper is version JBRIQE000000000.1.
